# The contribution of genetics and epigenetics to MAFLD susceptibility

**DOI:** 10.1007/s12072-024-10667-5

**Published:** 2024-04-25

**Authors:** Vittoria Moretti, Stefano Romeo, Luca Valenti

**Affiliations:** 1 Precision Medicine Lab, Biological Resource Center and Department of Transfusion Medicine, Fondazione IRCCS Ca’ Granda Ospedale Maggiore Policlinico Milano, Via F Sforza 35, 20122 Milan, Italy; 2 Department of Molecular and Clinical Medicine, The Sahlgrenska Academy, University of Gothenburg, Gothenburg, Sweden; 3 Department of Pathophysiology and Transplantation, Università degli Studi di Milano, Milan, Italy

**Keywords:** MAFLD, GWAS, Next-generation sequencing, Epigenetic factors, Therapeutic targets

## Abstract

Metabolic dysfunction-associated fatty liver disease (MAFLD) is the most common liver disease worldwide. The risk of developing MAFLD varies among individuals, due to a combination of environmental inherited and acquired genetic factors. Genome-wide association and next-generation sequencing studies are leading to the discovery of the common and rare genetic determinants of MAFLD. Thanks to the great advances in genomic technologies and bioinformatics analysis, genetic and epigenetic factors involved in the disease can be used to develop genetic risk scores specific for liver-related complications, which can improve risk stratification. Genetic and epigenetic factors lead to the identification of specific sub-phenotypes of MAFLD, and predict the individual response to a pharmacological therapy. Moreover, the variant transcripts and protein themselves represent new therapeutic targets. This review will discuss the current status of research into genetic as well as epigenetic modifiers of MAFLD development and progression.

## Introduction

Metabolic dysfunction-associated fatty liver disease (MAFLD) defines fatty liver disease related to systemic metabolic dysregulation due to insulin resistance [1]. MAFLD is a multifactorial disease that encompasses a spectrum of pathological conditions, ranging from simple steatosis (MAFL), MASH, and fibrosis/cirrhosis which can lead ultimately to hepatocellular carcinoma (HCC) [2]. This condition was previously known as non-alcoholic fatty liver disease (NAFLD), and is now also referred to as metabolic dysfunction-associated steatotic liver disease (MASLD) [3]. Between 1999 and 2022, MAFLD-related mortality rate increased from 0.2 to 1.7 per 100,000 individuals. Hence, it is estimated that the number of MAFLD death will rise along with the risk of type 2 diabetes (T2D) and cardiovascular disease [4]. People with MAFLD have metabolic dysfunction triggered by excess adiposity and insulin resistance, and features of the metabolic syndrome including dyslipidemia, hypertension, hyperglycemia, and pro-inflammatory state [5]. Indeed, a positive energy balance due to excess in food intake and sedentary lifestyle leads to excess adiposity and potentially ectopic lipid accumulation in the liver and insulin resistance [6]. Adipose tissue insulin resistance causes increased lipolysis with excess circulating free fatty acids (FFA) flux to the liver, while at the same time hyperinsulinemia promotes de novo lipogenesis in hepatocytes. When the ability of hepatocytes to get rid of excess lipids by secretion of lipoproteins and oxidation is overwhelmed, hepatic fat accumulation drives lipotoxicity, lipid peroxidation, and elevated reactive oxygen species (ROS) production [7]. In addition, intestinal dysbiosis and enhanced intestinal permeability with bacterial translocation into the liver contribute to inflammation [7]. In patients with MAFLD, T2D affects liver disease progression and advanced fibrosis leading to a major risk of adverse outcomes, and it is also associated with higher cardiovascular mortality [5].

**Fig. 1:**
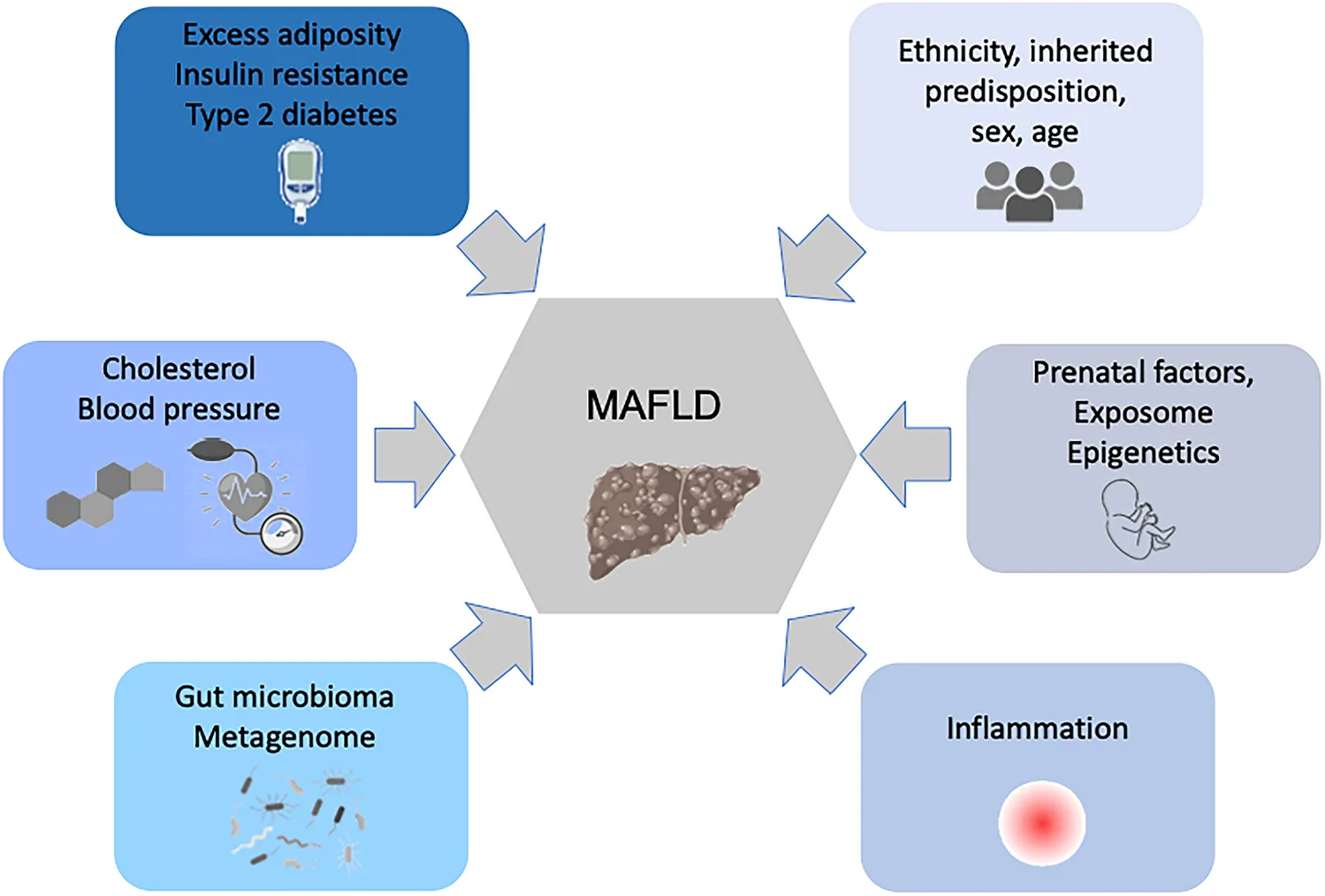
Heterogeneous factors lead to MAFLD, including ethnicity, sex, dietary habits, genetic predisposition, age, gut microbiota, and metabolic status. MAFLD is present if hepatic steatosis occurs with either obesity or overweight (BMI > 25 kg/m^2^ in white and >23 kg/m^2^ in Asian individuals), type 2 diabetes mellitus or evidence of metabolic dysregulation. At least two metabolic risk factors should be present for definition of metabolic dysregulation: waist circumference ≥102/88 cm in white male and female or ≥90/80 cm in Asian male and female; prediabetes; inflammation with elevated high-sensitive serum C-reactive protein level; elevated blood pressure or specific drug treatment; decreased HDL-cholesterol levels; increased plasma triglycerides levels. Heterogeneous factors lead to MAFLD, including ethnicity, sex, dietary habits, genetic predisposition, age, gut microbiota, and metabolic status

**Fig. 2:**
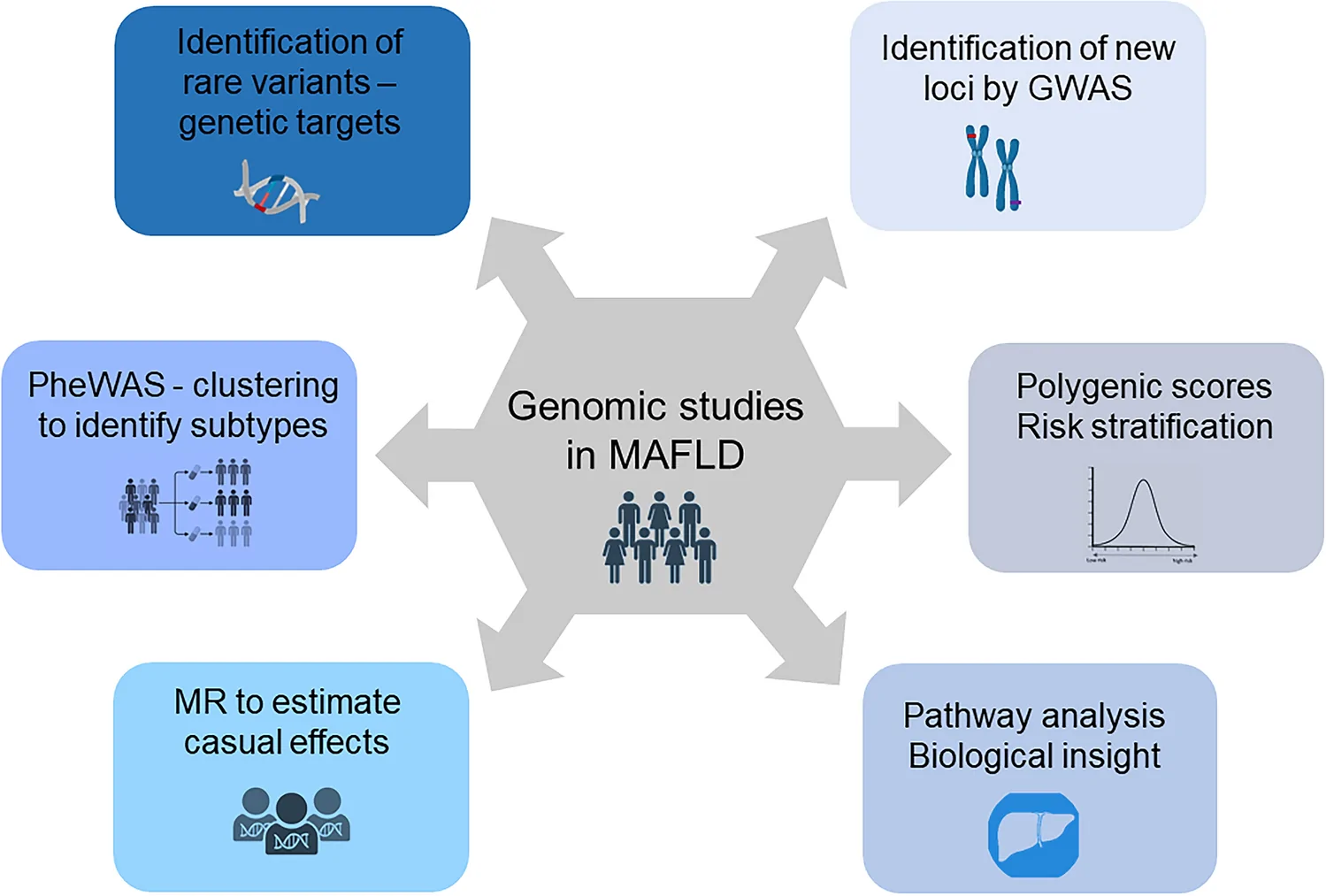
Genomic approaches to investigate MAFLD genetic determinants: GWAS to discovery the main common genetic variants; biological and pathways analysis to provide details regarding GWAS-prioritized tissues, and genes; PheWAS of MAFLD-risk-increasing alleles to identify distinct biological subgroupings; Mendelian randomization (MR) to estimate variant-MAFLD casual effect; PRS to stratify MAFLD risk in individuals with metabolic disorders; NGS techniques to identify rare variants involved in disease progression

The risk of developing MAFLD varies even among individuals with insulin resistance, due to a combination of environmental and inherited genetic factors. Environmental factors, such as smoking, air pollution, and the exposure to toxins, are implicated in the development of liver disease [7] (Fig. 1). In the last decades, major advances in genomics technologies and bioinformatics have led to a better comprehension of the causes behind liver disease [8]. Genome-wide association studies (GWAS) identified the main common genetic variants modulating hepatocellular lipid metabolism. However, all in all these loci explained less than 25% of disease heritability [9]. The molecular understanding of liver disease continues to be redefined since the introduction of “next-generation sequencing” (NGS) in clinical practice, which also permits to identify rare variants with a strong impact on protein function and to diagnose a considerable number of cases previously classified as “cryptogenic” [10, 11]. This review aims to provide an update of the current knowledge on MAFLD genetics and epigenetics. Taking into consideration that the majority of the literature focuses on NAFLD, we will extrapolate data from literature related to different analysis such as GWAS and whole-exome sequencing (WES) in NAFLD and discuss the recent discoveries and limitations of these approaches, including biological understanding, risk prediction, and drug development (Fig. 2).

## MAFLD heritability

Epidemiological studies in multi-ethnic cohorts, and familial and twin studies demonstrated that inherited factors play an important role in determining MAFLD predisposition [12, 13]. The risk of advanced fibrosis is more than 12-fold higher in first-degree relatives of patients with MAFLD cirrhosis than in those without MAFLD irrespective of metabolic triggers [12, 14]. Long et al. [15] demonstrated that a greater proportion of individuals with a parental history of MAFLD had hepatic steatosis as compared to those without MAFLD in parents. In keeping, twin studies led to the estimation that about 38 and 100% of hepatic fat content and MAFLD variability depend on inherited factors [14]. Advances in nuclear magnetic resonance techniques and in the measurement of hepatic fat and fibrosis by transient elastometry showed again that these traits are inherited for about 50% [13, 16]. Studies on multi-ethnic cohorts have demonstrated that there is a major inter-ethnic variability in MAFLD susceptibility which is high in people of Hispanic and East Asian ancestry, intermediate in Europeans, and lower in individuals with African ancestry [17, 18]. Interestingly, the rs738409 variant encoding for *PNPLA3* p.I148M accounts for a large fraction of the inter-ethnic variability in MAFLD predisposition (see below). However, a recent study suggests that the protection from chronic liver disease in African ancestry may be mostly accounted for by still unknown rare genetic variants [19, 20].

## Gene loci associated with MAFLD development by GWAS

In the last 15 years, GWAS allowed to identify the “low hanging fruit”, that is the main common genetic determinants of MAFLD [9, 21]. The first hits were highlighted in the patatin-like phospholipase domain-containing 3 (*PNPLA3*) [22], transmembrane 6 superfamily member 2 (*TM6SF2*) [23], glucokinase regulator (*GCKR*) [24], and membrane-bound O-acyl-transferase 7 (*MBOAT7*) genes [25]. Variants at these loci modulate lipid handling by hepatocytes, e.g., substrate delivery for de novo lipogenesis, formation of lipid droplets, utilization of lipid for mitochondrial energy, compartmentalization of fatty acid, catabolism, assembly of very low-density lipoprotein, and their secretion [26]. The *PNPLA3* p.I148M variant (rs738409 C>G) accounts for the largest fraction of genetic predisposition to MAFLD and it increases the susceptibility to the entire spectrum of hepatic damage associated with MAFLD, including MASH, fibrosis, and HCC [22, 27]. The presence of the p.I148M variant facilitates the accumulation of hepatic fat without affecting adiposity and insulin resistance [22] and it increases the risk of liver-related mortality in MAFLD patients and in the general population [28]. The carriage of rs58542926 C>T encoding for the p.E167K variant of *TM6SF2* also causes hepatic triglyceride accumulation in intracellular lipid droplets. *TM6SF2* is a transmembrane protein tightly bound to the endoplasmic reticulum, which stabilizes apolipoprotein B (ApoB) through two intraluminal loops [29]. The variant destabilizes the protein [29] hampering ApoB stability, lipidation, and secretion [30, 31]. The *TM6SF2* variant also predisposes to the full spectrum of liver damage, that is MASH, severe fibrosis, and HCC [23, 32], but at the same time protects against cardiovascular disease through the reduction in lipoprotein secretion [33]. The rs641738 C>T variant situated 500 bases-downstream of the *MBOAT7* gene is linked to fat deposition in the liver and the development of MAFLD, inflammation, fibrosis, and HCC [34]. The rs641738 variant is associated with lower hepatic mRNA and protein expression of MBOAT7, which is involved in the remodeling of phospholipids. The resulting increase in intracellular triglycerides is due to the induction a non-canonical pathway for triglyceride synthesis mediated by a futile cycle in phosphatidyl inositol metabolism [25]. The p.P446L *GCKR* variant (rs1260326) gene [24] curtails the ability to inhibit glucokinase in response to fructose-6-phosphate, thereby causing a constitutive activation of hepatic glucose uptake. This process reduces circulating glucose, but enhances the production of malonyl-CoA, which is a substrate for lipogenesis, blocking the oxidation of fatty acid and promoting fat accumulation in the liver [24].

In the last 2 years, recruitment of larger biobank cohorts led to pinpoint novel genetic determinants of MAFLD. Chen et al. [35] conducted a GWAS meta-analysis of MAFLD identified by imaging and diagnostic codes across diverse ancestries 17 new loci; the study highlighted new MAFLD-associated variants in or near fat mass and obesity associated (*FTO*), torsin family 1-member B (*TOR1B*), cordon-bleu WH2 repeat protein like 1 (*COBLL1*)/growth factor receptor-bound protein 14 (*GRB14*), insulin receptor (*INSR*), and sterol regulatory element-binding transcription factor 1 (*SREBF1*). The alteration in the expression of these genes affects insulin resistance, triglyceride, and cholesterol accumulation. By a phenome-wide association study (PheWAS), authors identified seven distinct clusters among the MAFLD variants and their associations with related phenotypes and cellular localization of the resulting protein product. In particular, alterations in *PNPLA2, INSR, SREBF1*, and *COBLL1* were grouped in “insulin resistance MAFLD subtype”; mutations in *GCKR* and *TRIB1* in “alteration of glucose level subtype”, variants in *GPAM*, *MARC1*, *TOR1B*, *ADH1B*, and *MBOAT7* in “triglyceride diversion/reduction subgroup”, *APOE* variants in “high/normal lipoprotein”, *FTO* variants in “low lipid burn subtype”, alterations in *MTTP* in “intestinal absorption” subgroup, and mutations in *PNPLA3, TM6SF2,* and *PTPRD* in “Low lipoprotein output” in subtype [35].

Furthermore, variants associated with cirrhosis trough alcohol consumption were found in alcohol dehydrogenase 1B (*ADH1B*), genes involved in de novo lipogenesis and retinol metabolism [35]. Moreover, Sveinbjornsson et al. [36] using magnetic resonance imaging, a meta-analysis of determinants of clinical MAFLD and integrating the results multiomics data pinpointed several genetic determinants as in a gene involved in lipogenesis, Apolipoprotein H (*APOH*) and cholesterol synthesis and Glucuronidase Beta (*GUSB*) which is related to glycosaminoglycan metabolism. Recently, a variant in the pleckstrin and Sec7 domain-containing 3 (*PSD3*) gene (rs71519934) was reported to reduce MAFLD susceptibility and it was associated with protection against MAFLD in individuals at risk. *PSD3* expression level is increased in MAFLD patients and its downregulation leads to a reduction in hepatocellular lipid content in mice and in several hepatocyte cell lines including human primary hepatocytes [37]. Gene variants associated with MAFLD predisposition are reported in Table 1.

**Table 1:** Germline variants associated with MAFLD development

Variant	Gene	Function	Effect	MAF	Phenotype
rs738409 C>G	*PNPLA3*	Lipid droplets	p.I148M	0.267	MAFLD, MASH, fibrosis, HCC
rs58542926 C>T	*TM6SF2*	VLDL secretion	p.E167K	0.074	MAFLD, MASH, fibrosis, HCC
rs1260326 C>T	*GCKR*	lipogenesis	p.P446L	0.293	MAFLD, protection against diabetes
rs641738 C>T	*MBOAT7/TMC4*	Lipid droplets	3’-UTR - p.G17E	0.388	MAFLD, MASH, fibrosis, inflammation, HCC
Several	*APOB*	VLDL secretion	Protein changes determining LoF	Rare	MAFLD, MASH, fibrosis, HCC
rs17817449 G>T	*FTO*	Adipogenesis	Intronic, c.46-30685T>A	0.392	MAFLD
rs7027757 G>A	*TOR1B*	TGL diversion	Intronic, c.465+49G>A	0.092	MAFLD
rs13389219 A>G	*COBLL1 – GRB1*	Adipogenesis	Intergenic	0.395	MAFLD
rs112630404 A>T	*INSR*	Insulin signaling	Intronic, c.653-33987A>T	0.148	MAFLD
rs4561528 C>T,G	*SREBP1*	Lipogenesis	Intergenic	0.383	MAFLD
rs140201358 G>C	*PNPLA2*	Lipid droplets	p.N252K	0.013	MAFLD
rs28601761 G>C	*TRIB1*	Insulin signaling	Intergenic	0.413	protection against MAFLD
rs2792751 T>C	*GPAM*	Lipogenesis	p.I43V	0.269	MAFLD
rs2642438 A>G	*MARC1*	TGL diversion	p.T165A	0.291	protection against MAFLD
rs1229984 T>C	*ADH1B*	TGL diversion	p.H48R	0.031	protection against MAFLD
rs429358 C>T	*APOE*	Lipoproteins	p.C130R	0.154	MAFLD
rs10756038 A>G,T	*PTPRD*	Lipid droplets	Intronic, c.-599-121406T>C	0.300	MAFLD
rs1801689 C>A	*APOH*	Lipoproteins	p.C325G	0.020	MAFLD
rs6955582 A>G	*GUSB*	Lysosomes	Intronic, c.1653+1032T>C	0.449	MAFLD
rs71519934 GT>AG	*PSD3*	Lipogenesis	p.T186L	0.330	protection against MAFLD
Several	*MTTP*	TGL diversion	Protein change	Rare	MAFLD

## Polygenic risk score for MAFLD risk prediction

Polygenic risk scores (PRS) were developed to summarize the effect variants for MAFLD, such as those in *PNPLA3–TM6SF2–GCKR–MBOAT7*, e.g., the hepatic fat PRS, or PRS-HFC, and then adjusted for a protected variant in and 17-β hydroxysteroid dehydrogenase 13 (*HSD17B13*) (PRS-5) [38–40], to improve MAFLD risk stratification. These PRS allowed to demonstrate that genetic predisposition derived by rare combinations of common variants contribute to severe or early onset liver disease phenotypes [38]. The integration of genetics with clinical fibrosis scores refines individual risk and prediction for liver disease, mainly in subjects at risk for MAFLD. PRS provide evidence that common genetic variants capture additional prognostic insights not showed by validated clinical/biochemical parameters [40]. Moreover, PRS improve the accuracy of HCC detection and may help stratify HCC risk in individuals with dysmetabolism, including those without severe liver fibrosis [38].

## Rare variants predisposing to MAFLD

In the last 5 years, the advent of NGS studies has led to the identification of genes whose rare loss-of-function (LoF) variants most frequently contribute to MAFLD. Rare variants in Apolipoprotein B (*APOB*) predispose to MAFLD and they are responsible for the development of severe MAFLD and hypobetalipoproteinemia. Familial hypobetalipoproteinemia (FHBL) is a codominant disorder of lipoprotein metabolism derived from mutations in the *APOB* gene encoding for ApoB and characterized by low levels of LDL-cholesterol. FHBL can be caused by truncations of full-length ApoB-100 as short as 5% (apoB-5) and as long as 89% (ApoB-89) [33, 41, 42]. Rare variants in *MTTP* gene have been linked with susceptibility to MAFLD and rare and loss-of-function mutations in *MTTP* result in abetalipoproteinaemia. The gene encodes for microsomal triglyceride transfer protein (MTP) which forms a heterodimer with protein disulfide isomerase (PDI), and catalyzes the lipidation and assembly of ApoB [43]. These data suggest that lipoprotein retention in hepatocytes plays a key role in MAFLD. In addition, rare LoF mutations in autophagy related 7 (*ATG7*) gene modulating lipophagy and mitophagy in hepatocytes have been implicated in disease progression [44].

## Gene loci associated with MAFLD progression

Through the evaluation as outcomes of severe liver disease phenotypes, GWAS have also led to the discovery of genetic variants implicated in MAFLD progression to steatohepatitis, fibrosis, and cirrhosis, besides in hepatic fat accumulation per se. The mechanisms are related to interferences with oxidative stress, cell senescence, fibrogenesis, insulin signaling, glucose metabolism, inflammation, and lipotoxicity [45]. Oxidative stress and deranged mitochondrial respiratory complex activity and oxidation, namely mitochondrial dysfunction, are considered a main contributor to liver injury and MAFLD progression [46]. Indeed, among the determinants of liver damage and cirrhosis risk in Europe are Homeostatic Iron Regulator (*HFE*) p.C282Y and p.H63D variants (rs1800562, rs1799945) associated with hemochromatosis, type 1 [47]. Excess tissue iron predisposes to the development and progression of MAFLD by catalyzing oxidative stress [48]. Other genes whose mutations are implicated in iron disorders and in MAFLD are ceruloplasmin (*CP*), serpin family A member 1 (*SERPINA1*), and proprotein convertase subtilisin/kexin type 7 (*PCSK7*), which is involved in hepatic inflammation by modulating multiple pathways, such as lipid, iron metabolism, and fibrogenesis [44, 49, 50]. Other pathways are also involved. For example, variants in Fibronectin Type III Domain Containing 5 (*FNDC5*) have been linked with fibrosis progression: the gene encodes for a myokine named Irisin involved in hepatic stellate cell activation and fibrogenesis [51, 52]. Moreover, concerning hepatic inflammation, two variants in interferon *IFN-λ3/IFN-λ4* region in linkage disequilibrium among them were confirmed to be associated with more severe fibrosis in MAFLD by modulating the activation of innate immunity and inflammation [53, 54]. Alterations in insulin signaling lead to a more severe fibrosis: examples are variants in Ectonucleotide Pyrophosphatase/Phosphodiesterase 1 (*ENPP1*), Insulin Receptor Substrate 1 (*IRS1*), and Tribbles homolog 1 (*TRIB1*) [55, 56]. The main modulator of steatohepatitis affects *HSD17B13*, a lipid droplet (LD)-associated protein that is mainly expressed in hepatocytes. LoF variants (mainly rs72613567: TA) of this gene mitigate the progression of MAFLD, reducing the risk of steatohepatitis, cirrhosis, and HCC. Genetic variants in *HSD17B13* result in a loss of expression and/or of enzymatic activity, toward retinol, steroid hormones, and other pro-inflammatory lipid mediators, and increase retinol–retinol binding protein (RBP4)–transthyretin (TTR) transport from hepatocytes [57]. However, despite this evidence, the function of *HSD17B13* and how its absence protects against MASH remains obscure. Finally, concerning the role of rare variants, those in *ATG7* were identified as modifiers of MAFLD progression in Europeans by enhancing specifically the risk of hepatocellular ballooning (e.g., rs143545741 C>T and rs36117895 T>C) [58], as well as LoF mutations in Telomerase Reverse Transcriptase (*TERT*) were associated with liver senescence and development of HCC [59]. Variants/genes modulating MAFLD progression are presented in Table 2.

**Table 2:** Germline variants associated with MAFLD progression

Variant	Gene	Function	Effect	MAF	Phenotype
rs72613567 T>TA	HSD17B13	Retinol, steroid and lipid metabolism	Splice c.704+2dup	0.250	protection against MAFLD, fibrosis, cirrhosis and HCC
rs762623 G>A	CDKN1A	modulation of cell cycle regulator p21	Intronic, c.-141-7G>A	0.118	fibrosis and in cell senescence
rs3480 A>G	FNDC5	hepatic stellate cells activation	Intronic, c.*1730C>T	0.565	fibrosis
Several	TERT	cell senescence	Protein change	Rare	Fibrosis, cell senescence, HCC
rs12979860 C>T	IFN-λ4	activation of innate immunity and necroinflammation	Intronic, n.429-152G>A	0.310	fibrosis
rs236918 G>C	PCSK7	sTfR generation and iron homeostasis	Intronic, c.1156-1135	0.154	Dyslipidemia, fibrosis, iron overload
rs1044498 A>C	ENPP1	Insulin signaling	p.K121Q	0.177	fibrosis
rs1801278 G>A	IRS1	Insulin signaling	p.G972R	0.062	fibrosis
rs1800562 A>G; rs1799945 C>G	HFE	Iron metabolism	p.C282Y; p.H63D	0.038; 0.099	Iron overload, MAFLD
Several	CP	Iron metabolism	Protein change	Rare	Iron overload, MAFLD
rs28929474 G>A	SERPINA1	ER stress, iron metabolism	p.E366K	0.013	Iron overload, MAFLD
rs143545741 C>T; rs36117895 T>C	ATG7	Autophagy	p.Pro426Leu; p.V471A	0.001; 0.036	MAFLD
rs4374383 G>A;	MERTK	Fibrogenesis	Intronic, c.2079+3127A>G	0.579	MAFLD, fibrosis
rs3750861 G>A	KLF6	Regulation of de novo lipogenesis fibrogenesis	Intronic, c.103-27G>A	0.077	MAFLD

## Sex-genotype epistasis in MAFLD

**Fig. 3:**
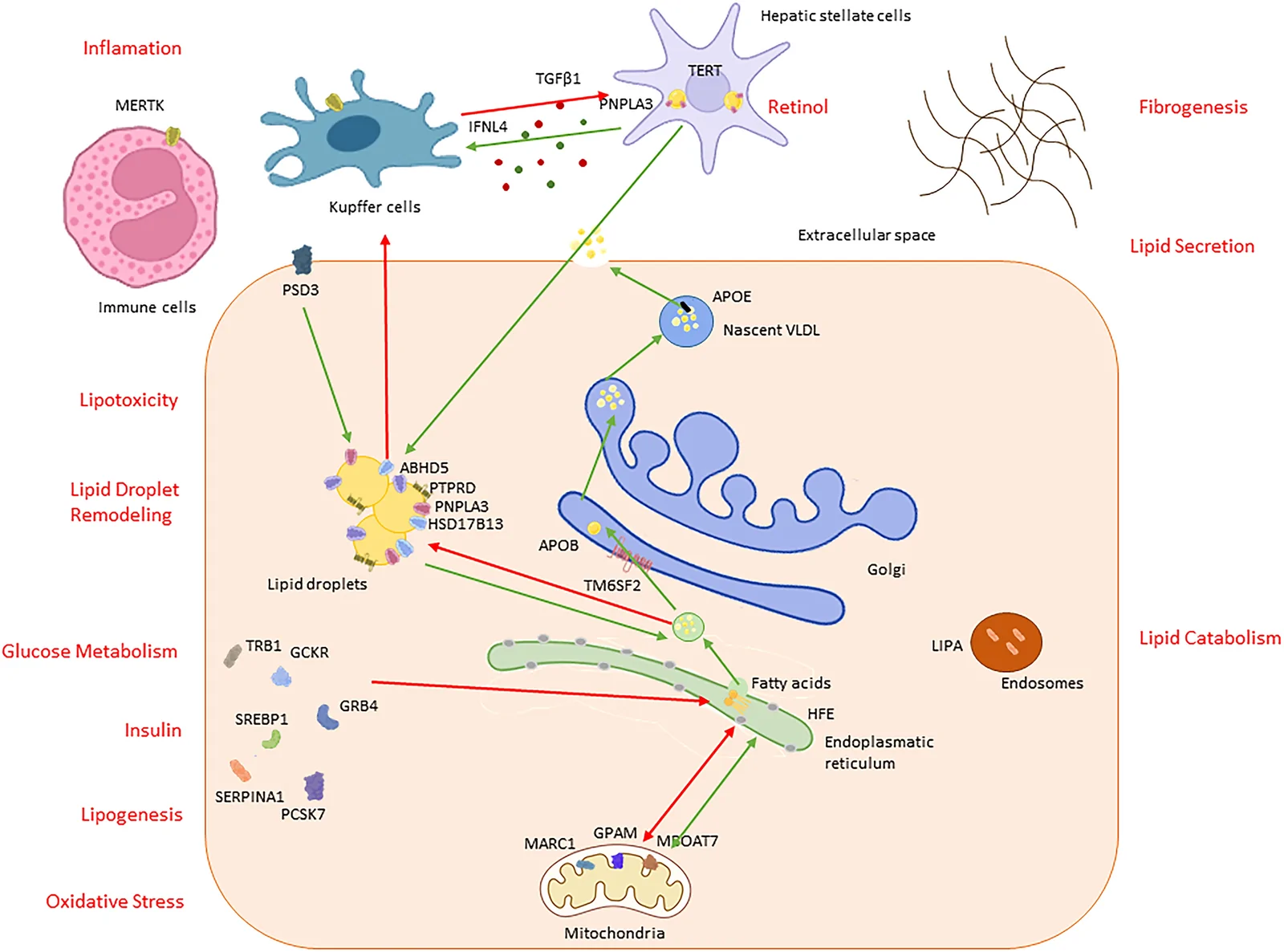
Genetic determinants of MAFLD, classified according to the biological processes by which the encoded proteins are thought to contribute to the pathogenesis. Red arrows indicate pathological processes/lipid fluxes, whereas green arrows indicate beneficial pathways. Pathophysiological processes are indicated in red, while genes, and cellular and liver compartments in black

Excess adiposity is a main trigger of MAFLD genetic susceptibility [58, 60]. Sex hormones have also a major role in modulating liver fat content [61]. Estrogens protect against MAFLD, accounting for a lower prevalence of this condition in female before menopause. In older male, lower testosterone levels are associated with frailty and increased risk of MAFLD, while, in both sexes, lower sex hormone-binding globulin (SHBG) circulating levels are associated with MAFLD [62]. High androgen levels in female with polycystic ovary syndrome (PCOS) lead to a markedly increased risk of MAFLD, as well as insulin resistance and obesity [63]. Recently, Cherubini et al. [64] demonstrated that there is an interaction between sex and the common genetic variant *PNPLA3* p.I148M in determining the development and severity of MAFLD. This relation, documented both at the epidemiological and a molecular level, contributes explaining why a subset of Female develop rapidly progressive MAFLD at menopause. Indeed, estrogens protect premenopausal female against MAFLD operating on lipid metabolism at a systemic level and in hepatocytes through estrogen receptor alpha (ERα) [65]. Instead, following menopause, lower estrogens cannot inhibit de novo lipogenesis, favoring import and accumulation of lipid in the liver [64]. Postmenopausal female carrying of *PNPLA3* p.I148M variant has a persistent induction and accumulation of the mutant PNPLA3 protein in hepatocytes and consequently enhanced hepatic fat accumulation and fibrogenesis [64] (Fig. 3).

## Acquired somatic variants in MAFLD

Somatic mutations are non-heritable gene variants that occur in aged or chronically injured somatic cells, and this phenomenon is also observed in livers from individuals with MAFLD. The acquisition of somatic variants and clonal expansion leads to the progression of chronic liver diseases into HCC [66, 67]. Somatic alterations occurring in genes involved in lipid metabolism are also implicated in MAFLD progression to cirrhosis. Indeed, in individuals with the most advanced liver disease, there are evidences of selection of somatic variants in Forkhead Box O1 (*FOXO1*), Cell Death Inducing DFFA Like Effector B (*CIDEB*) and Glycerol-3-Phosphate Acyltransferase, Mitochondrial (*GPAM*), involved in the regulation of lipid synthesis and the antioxidant response, leading to a reduction of hepatic fat accumulation possibly accounting for “burnt-out steatohepatitis”, as an adaptive response against chronic lipotoxicity [68, 69]. On the other hand, somatic variants accumulation occurring in genes involved in chromatin remodeling [i.e., AT-Rich Interaction Domain 1A (*ARID1A*), AT-Rich Interaction Domain 2 (*ARID2*), and Lysine Methyltransferase 2C (*KMT2C*)] and in cell differentiation and migration (i.e., RAS, MAPK, AKT, mTOR, and MET pathways) contribute to hepatic inflammation and carcinogenesis [67, 70]. Importantly, while germline common and rare variants affect primarily hepatocyte triglyceride homeostasis and inflammation that are the initial disease stage, the somatic ones are mostly present in the late stage.

In regards to HCC, it is not surprising that somatic mutations in *TERT* promoter account for 60% of HCC cases [71]. Alterations in *TERT* promoter cause the reactivation of telomerase reverse transcriptase causing telomere re-elongation and immortalization of the neoplastic clone; these variants occur not only in cancer tissue but also in early cirrhotic tissue (6–19%) highlighting their importance in the disease progression and tumorigenesis [72]. Similarly, another frequent gene affected by somatic variants is Tumor Protein P53 (*TP53*) (45% of HCC cases), a tumor suppressor protein involved in the maintenance of genome integrity inducing cell cycle arrest, apoptosis, and senescence in response to cellular stress [73]. Aberrant reactivation of Wnt-β-catenin pathway due to somatic alterations in Catenin Beta 1 (*CTNNB1*) is present in 30% of the cases, while for 10% of the cases mutations involved Axin 1 (*AXIN1*) gene [74, 75]. Furthermore, recent studies have shown an excess of somatic variants predisposing to clonal haematopoiesis of indeterminate potential (CHIP), a precursor of hematologic cancer, in individuals with MAFLD. Somatic mutations in Tet Methylcytosine Dioxygenase 2 (*TET2*), ASXL Transcriptional Regulator 1 (*ASXL1*), and Janus Kinase 2 (*JAK2*) genes are implicated in CHIP [76]. In general CHIP may induce chronic liver disease progression via an aberrant inflammatory response [77, 78]. Also, CHIP is relatively common in patients with solid tumor malignancies and it is associated with adverse outcomes of hematologic malignancies. However, it is fair to say that the risk associated with CHIP for progressive MAFLD is negligible as compared to blood cancer. Several examples of genes in which somatic variants occur are reported in Table 3.

**Table 3:** Genes involved in somatic variants implicated in MAFLD and HCC

Gene	Function
ARID1A, ARID2, KMT2C, SETD2, KMT2D, PBRM1, SMARCA1, SMARCA2, SMARCA4, KMT2B, DNMT3A, ASXL1, TET2	Chromatin remodeling pathway
TP53, FOXD4, CDKN2A, PTPRB, ATM, IRF2, RB1, TSC1, MDM2, ADAMTS9	Cell cycle control
CTNNB1, AXIN1, CDH8,APC	WNT/β-Catenin pathway
RPS6KA3, PREX2, PI3CA, PTEN, PTPN13, IL6ST, MET	PI3K/RAS pathway
FOXO1, CIDEB, ACVR2A, LRP1B, APOB, ALB, GPAM, PLCB4, TNRC6B, FAT4, HNF1	Lipid metabolism
TERT, NEAT1, MUC21, BRCA2, NOTCH3, HLA-F, BRCA1, TPRXL, MYD88, TSC2, NFE2L2, CAMTA1 ZNF521, PROKR2, KRAS, CSMD3, FAT3, KEAP1, SF3B1, VEGFA	Cancer development and progression
DNMT3A, ASXL1, SRSF2, RUNX1 PPM1D, CBL, CUX1, BCOR, BCORL1, GNAS, GNB1, U2AF1, TET2	Clonal hematopoiesis

## Epigenetic alterations in MAFLD

Epigenetic factors are stable modifications of chromosomes/DNA that modify gene expression and cause phenotypic variation without direct alteration of DNA base sequence. Epigenetic alterations encompass DNA methylation, histone modifications, and modulation of gene expression by microRNAs (miRNA) and other non-coding RNAs [79]. DNA methylation occurs when methyl groups are covalently bound to cytosine to produce 5-methylcytosine near to guanine (CpG dinucleotides), which is most frequently located at the promoter region of genes. This reaction is catalyzed by DNA methyltransferases (DNMTs). Hypermethylation of CpG islands is associated with gene silencing, while hypomethylation leads to gene activation [79]. Some studies have reported a role of global hypomethylation and differential methylation in the progression of MAFLD, highlighting also specific methylation shifts at transcriptional start of genes involved in lipid metabolism and energy homeostasis. There is evidence of changes in methylation of genes regulating lipid and cholesterol transport (APO family members and STARD) and the metabolic hormone fibroblast growth factor 21 (*FGF21*), which is high expressed in the liver and modulates systemic energy values acting in macronutrient metabolism [80]. Pirola et al. [81] showed that silencing of mitochondrial gene NADH dehydrogenase 6 (MT-ND6) by promoter hypermethylation correlated with MAFLD severity. Furthermore, hypermethylation of the hepatic promoter of the peroxisome proliferative activated receptor (*PPAR*)-gamma coactivator one alpha (*PGC1*-α) gene, a transcriptional regulator of mitochondrial fatty acid oxidation was associated with peripheral insulin resistance and fasting insulin levels of MAFLD patients [82]. Two studies highlighted a general hypomethylation status of hepatic DNA in patients with MAFLD compared to individuals with healthy liver, and a more marked demethylation in patients with advanced compared to milder MAFLD [83, 84]. Conversely, *PNPLA3* was reportedly hypermethylated in patients with MAFLD [85], but evidence is controversial and specific hypomethylation may be linked to higher gene expression in people carrying at risk genotypes and in female [64, 86]. GWAS analyses evidenced that hypomethylated loci are near to genes involved in cancer and immunoresponse, whereas hypermethylated regions occur close to genes associated with lipid metabolism [87, 88]. Another epigenetics modification consists of the addition of methyl groups, acetyl groups or phosphoryl groups to histone proteins leading to an alteration of the physical structure of chromatin and changing the ability of recruitment of other proteins. Some studies reported a correlation with aberrant histone methylation and acetylation profiles and metabolic syndrome and alteration in the expression of specific histone lysine methyltransferases (*KMT*) and demethylases (*KDMs*) during MAFLD [89, 90]. Histone acetyltransferases (*HATs*) promote gene transcription, by increasing the accessibility to DNA, whereas histone deacetylase (*HDACs*) repress it. The HAT p300 activates the glucose-responsive lipogenic activators ChREBP promoting the lipogenesis and steatosis. Conversely, Histone Deacetylase 3 (*HDAC3*), Sirtuin 1 (*SIRT1*), and Sirtuin 6 (*SIRT6*) protect against MAFLD by deacetylating promoter histones of lipogenic genes [91, 92]. Alterations in miRNA expression have also been associated with MAFLD development and progression. miRNAs are a class of endogenous non-coding functional RNAs implicated in the regulation of gene expression by interacting with complementary non-coding regions of genes and other RNAs. Cell death and degeneration during MAFLD lead to the release of different miRNAs which can regulate an array of biological processes, such as lipid metabolism, glucose catabolism, inflammation, cell proliferation and apoptosis, adipocyte differentiation, and insulin resistance [93, 94]. Alterations in miRNAs involved in the regulation of hepatic cholesterol and lipid metabolism contribute to the development of metabolic disorder, atherosclerosis, and cardiovascular disease. In particular, miR-122 has been causally involved in MAFLD development: it represents the 70% of hepatic miRNAs and it plays an important role in the regulation of genes associated with liver regeneration, lipid, and cholesterol metabolism. Lower levels of miR-122 in hepatocytes are implicated in the activation of fibrotic pathways and in the reduction of lipid secretion [95]. Conversely, circulating levels of miR-122 are increased in patients with compared to those with simple steatosis and general population, due to the release of circulating miRNAs from hepatocytes [96]. Also, miR-192, and miR-375, miR-19a/b and miR-125b, which are overexpressed in subjects with MAFLD, participate in the development of MASH. Specifically, miR-192 is induced by TGFβ1 contributing to fibrosis development, miR-375 regulates glucose levels, and the requirement for adaptive β cell expansion, while miR-19a/b related to NF-κB signaling and miR-125b are associated with cardiovascular disease and inflammation [93]. Also, miR-10b, miR-144, miR-146b, and miR-155 participate in hepatic inflammation and liver damage by regulating PPAR-α, Toll-like receptors (TLR), and Tumor Necrosis Factorα (*TNFα*) [97]. Another risk factor for MAFLD development is the upregulation of miR-29 a/b/c which leads to insulin resistance through the block of Akt pathway and insulin signaling [98]. Yu et al. [99] showed that overexpression of miR-33a/b in hepatocytes determines triglycerides accumulation and promote steatosis, whereas overproduction of miR-34 a/b/c promotes lipid metabolism by targeting acyl-CoA synthetase long-chain family member 1 (*ACSL1*) [100]. Recently, a role of miR-21 in the transition to HCC was reported, mediated by silencing of HMG box-containing protein 1 (*HBP1*) and of the consequent activation of p53 [93]. Apart from these, other miRNAs reportedly dysregulated in MAFLD are shown in Table 4.

**Table 4:** miRNAs dysregulated in MAFLD

microRNA	Dysregulation	Disease output
miR-9, miR-16, miR-23a, miR-27b, miR-30c, miR-31, miR-101, miR-103, miR-106, miR-107, miR-125b, miR-144, miR-149, miR-150, miR-152, miR-181a, miR-182, miR-183, miR-192, miR-194, miR-200a/b/c, miR-212, miR-214, miR-223, miR-224, miR-291b, miR-301a-3p, miR-331, miR-335, miR-375, miR-378, miR-421, miR-429, miR-892a, miR-1282, miR-1290, miR-3663-5p, miR-3924, miR-451	Upregulation	MAFLD
miR-17, miR-26, miR-27a, miR-29a/c, miR-30b, miR-99a, miR-139-5p, miR-146b, miR-181d, miR-197, miR-198, miR-203, miR-378i, miR-422a, miR-467b, miR-576, miR-590, miR-451	Downregulation	
miR-15b, miR-19a/b, miR-24, miR-33a/b, miR-34 a/b/c, miR-122, miR-21	Upregulation	Lipid synthesis and accumulation
miR-216, miR-302a, miR-122, miR-199a-3p	Downregulation	
miR-221, miR-222, miR-219a	Upregulation	Fibrosis
miR-21, miR-155	Upregulation	HCC
miR-601, miR-617, miR-641, miR-765	Downregulation	MASH
miR-155, mir-223	Upregulation	Insulin signaling
miR-10b	Upregulation	Hepatic inflammation
miR-143	Downregulation	

## Conclusion

**Fig. 4:**
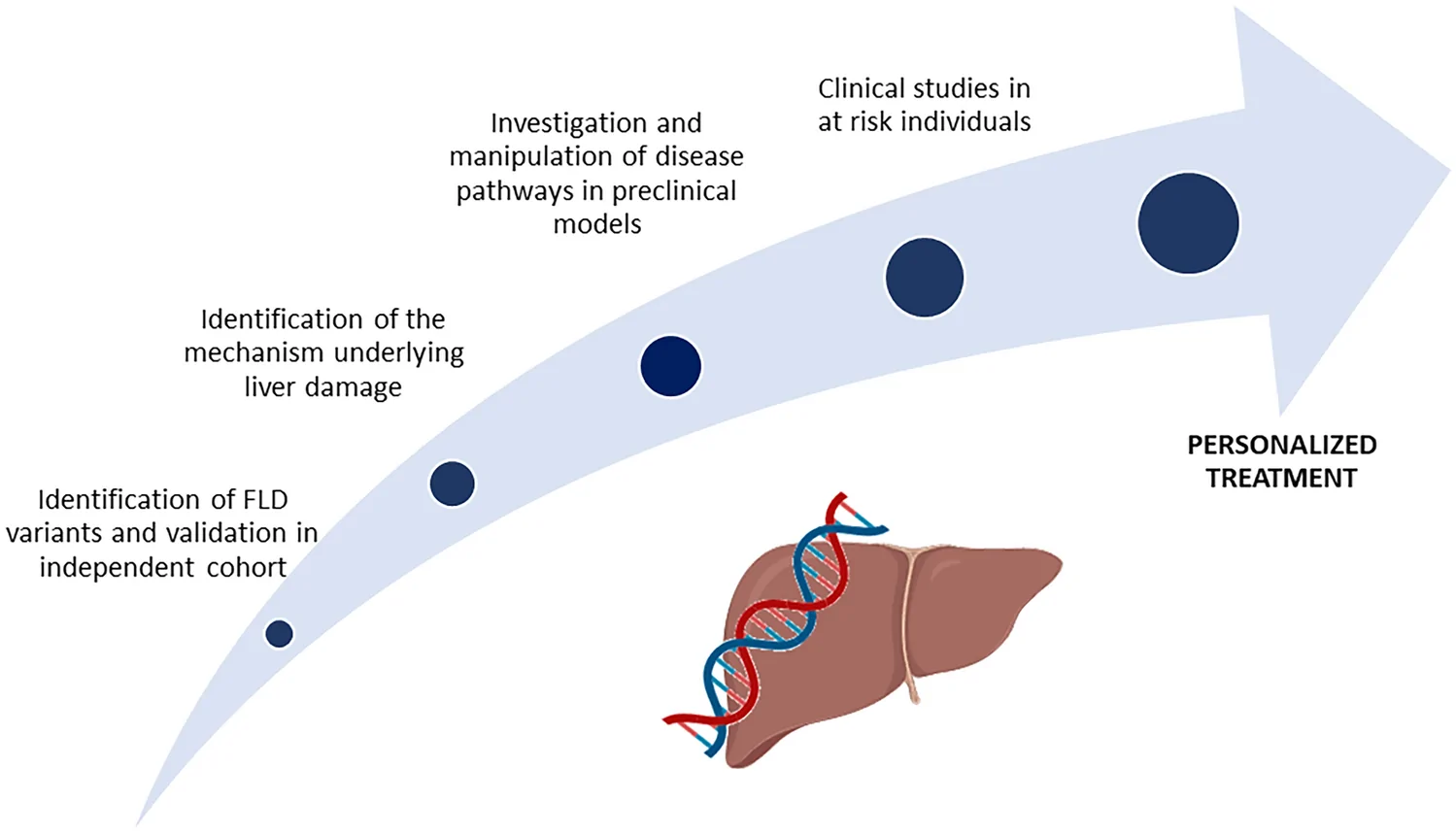
Genomic studies to develop new MAFLD therapeutics and precision medicine approach

Genetic variation plays a key role in determining the susceptibility to the development and progression of MAFLD extending to liver-related disease and overall mortality. Genetic determinants have an effect size comparable and synergic to that of the main metabolic risk factors, such as obesity and type 2 diabetes. Thanks to genomic studies subsets of patients with different pathophysiology, risk of liver-related complications and response to treatment can now be profiled. In the coming years, the search of genetic mutations will continue increasing the number of individuals in GWAS and introducing novel strategies as Mendelian randomization methods and meta-analysis allowing to explore the reasons for heterogeneity of the genetic effects across datasets. The high gene x environment interactions observed in the genetic architecture of MAFLD, and the rise of prevalence of people at risk (i.e., obese, insulin resistant) and with severe disease may lead to the identification of new loci and precision medicine strategies. In conclusion, genomic studies are revolutionizing the comprehension of MAFLD leading the way to new tools for targeted screening of high-risk individuals, also improving patient stratification for clinical trials, for prognostication and clinical management (Fig. 4).

## Data Availability

This is a review article utilizing data from published papers and other public database and, as such, data availability statement is not applicable.
